# Beyond immunity: macrophage plasticity in vascular regeneration

**DOI:** 10.1038/s44385-026-00114-5

**Published:** 2026-09-24

**Authors:** Si Tong Huo, Taishi Inoue, Clinton S. Robbins, Sara S. Nunes

**Affiliations:** 1 University Health Network Research Institutes, Toronto, ON Canada; 2 Institute of Biomedical Engineering, University of Toronto, Toronto, ON Canada; 3 Laboratory of Medicine and Pathobiology, University of Toronto, Toronto, ON Canada; 4 Peter Munk Cardiac Centre, Toronto, ON Canada; 5 Ajmera Transplant Centre, University Health Network Research Institutes, Toronto, ON Canada; 6 Heart and Stroke/Richard Lewar Centre of Excellence, University of Toronto, Toronto, ON Canada

**Keywords:** Cell biology, Immunology, Stem cells

## Abstract

Macrophages drive microvascular development, repair, and remodeling in addition to their roles in immunity. Single-cell transcriptomics reveals macrophage subpopulations whose vascular functions exceed the M1/M2 framework. Here we review four mechanisms of macrophage-driven angiogenesis—paracrine secretion, endothelial contact, matrix remodeling, and mural cell crosstalk—and their temporal ordering across development, injury, and disease. Finally, we examine translational strategies and propose a stage-resolved framework for driving revascularization or suppressing tumor angiogenesis.

## Introduction

Macrophages are a vital component of the immune system, traditionally known for their roles in phagocytosis and pathogen clearance. Beyond immunity, they are now also implicated in tissue homeostasis and repair, cancer, metabolic disease, fibrosis, angiogenesis, and vascular remodeling^1,2^. The advent of single-cell RNA sequencing (scRNA-seq) has transformed our understanding of macrophage heterogeneity, revealing transcriptionally distinct subpopulations with organ-specific identities and specialized vascular functions that far exceed what the classical M1/M2 framework could capture.

Macrophages interact with blood vessels in tissue-specific ways, including vessel-associated macrophages in the dermis^3^ and perivascular macrophages in the brain^4^ that exhibit specialized functions in vascular integrity, angiogenesis, and tissue homeostasis. One of the earliest descriptions of perivascular macrophages dates to the 1930s, when Castaneda observed their presence at sites of tissue injury^5^. Since then, advances in high-resolution transcriptomics have revealed that perivascular macrophages constitute a heterogeneous family of cells defined by organ context, developmental origin, surface markers, and functional state. Lapenna et al. provided a comprehensive review of distinct perivascular macrophage populations and their functions in health and disease^3^, highlighting their roles in regulating vessel permeability, immune surveillance, steroidogenesis in health, as well as angiogenesis in tumor growth and various roles in the central nervous system.

Beyond their tissue-resident roles, macrophages also contribute to microvascular repair and regeneration. After tissue injury, macrophages accumulate at sites of vascular damage and coordinate capillary formation and small vessel restoration, processes central to the treatment of ischemic heart disease, peripheral artery disease, and other vascular disorders. Current vessel regeneration approaches include tissue-engineered vascular grafts^6^, stem cell therapies^7^, and bioengineered or 3D printed scaffolds^8^, with the first FDA approval of an acellular tissue-engineered vessel (Symvess) in December 2024 for large vessel applications. However, challenges at the microvascular level remain, including limited long-term graft survival, insufficient vascularization of engineered constructs, and production costs that restrict clinical translation. While endothelial cells and pericytes have been the focus of vascular biology, macrophages are now recognized as orchestrators of blood vessel formation, stabilization, and remodeling. The macrophage subpopulations that mediate these processes, their transcriptional identities, ontogenies, and context-dependent behaviors, remain incompletely defined.

This review addresses the molecular mechanisms by which macrophages drive vessel formation: stage-specific growth factor secretion, direct interactions with endothelial cells, extracellular matrix(ECM) remodeling, and crosstalk with mural cells. We then describe how these mechanisms are conserved across development, wound repair, and cancer. Finally, we consider how this stage-resolved view maps onto current therapeutic strategies and early-phase clinical trials.

## Macrophage phenotypic spectrum and environmental regulation in vascularization

Macrophages in vascularized tissues derive from two sources: embryonic progenitors in the yolk-sac seed tissues before birth, which give rise to self-renewing resident populations that persist into adulthood; and circulating monocytes generated in the bone marrow are recruited across the vessel wall and differentiate locally^1,2^. These are not sealed compartments. Across multiple organs, tissue-resident macrophages segregate into at least three coexisting subsets: TIMD4/LYVE1/FOLR2-expressing (TLF+) self-renewing macrophages, CCR2+ monocyte-replenished macrophages, and MHC-II^hi^ macrophages with distinct life cycles^9^. Origin, however, does not assign function. Neither origin maps onto the inflammatory-reparative division that the M1/M2 framework implies, which itself is oversimplified because macrophage phenotype is continuously updated by local metabolic, mechanical, and immunological cues^10^. scRNA-seq profiling of monocyte-derived macrophages across 12 tissues and 25 biological conditions identified four conserved activation trajectories: phagocytic, inflammatory, oxidative stress, and remodeling, indicating that macrophages of identical ontogeny diverge dramatically in response to local context^11^. Recruited macrophages are better described as inflammatory on arrival than inflammatory by identity: in wound repair they mature in situ into a long-term resident, alternatively activated population, and it is failure of that conversion, rather than the recruitment itself, that sustains inflammation in impaired healing (Section “Clinical implications and therapeutic potential”). Resident macrophages are correspondingly not intrinsically reparative, and mount pro-inflammatory programs when the local milieu demands it^10^. The pro- and anti-angiogenic output of a given macrophage population therefore cannot be inferred from lineage alone but is set by the signals received from the local environment.

**Fig. 1:**
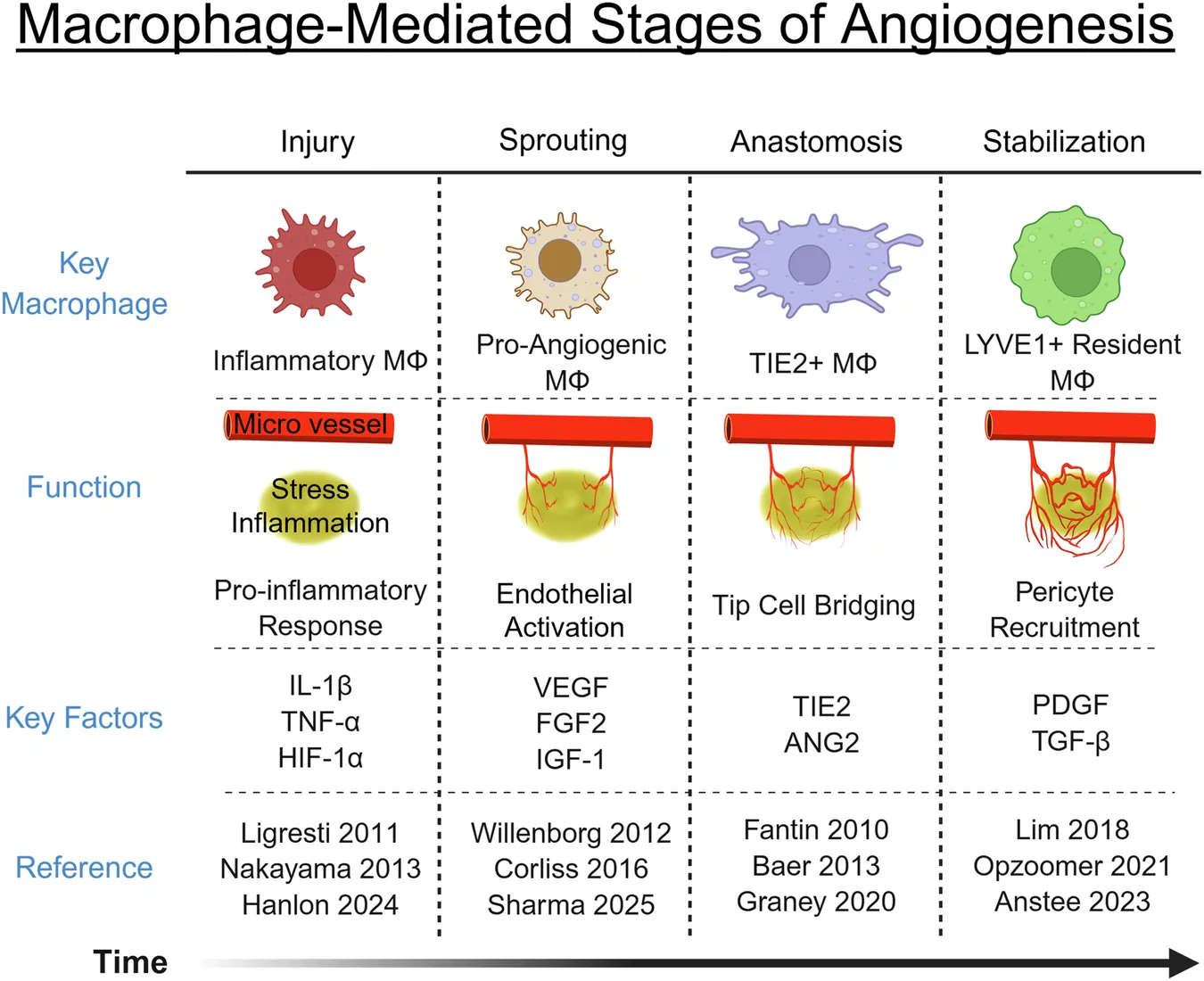
Temporal coordination of macrophage subpopulations across successive stages of angiogenesis. Columns represent 4 sequential stages of the angiogenic response and their predominant macrophage type, ordered left to right along the time arrow: injury, sprouting, anastomosis and stabiliztaion.

Several microenvironmental signals shift macrophage phenotype along the angiogenic spectrum, with direct consequences for microvascular outcome^12–21^ (Fig. 1).

The cytokine milieu is a primary determinant of macrophage angiogenic phenotype; different cytokines drive macrophages into distinct activation programs with distinct vascular consequences. TGF-β1, released in the remodeling microenvironment, skews macrophage polarization toward a pro-angiogenic phenotype through SNAIL upregulation downstream of SMAD2/3 and PI3K/AKT signaling, resulting in increased expression of VEGFA, CD206, and CXCR4 and suppression of pro-inflammatory markers^22^. IFN- γ imposes the opposite output: it promotes pro-inflammatory antimicrobial phenotypes in synergy with LPS via NF-κB target genes^23^. The anti-angiogenic VEGF165b isoform locks macrophages into a pro-inflammatory state via S100A8/S100A9-mediated inhibition of VEGFR1-STAT3 signaling, impairing muscle revascularization in peripheral artery disease. Restoring pro-angiogenic polarization by neutralizing VEGF165b rescues perfusion recovery, directly linking cytokine-driven macrophage phenotype to microvascular outcome^24^. Ligand identity, however, is not the only variable: the same receptor can be read differently depending on which ligand engages it. CSF-1R is the clearest illustration, being unusual among growth factor receptors in having two structurally unrelated cognate ligands, CSF-1 and the more recently identified IL-34. The two are equivalent in their ability to drive monocyte-to-macrophage differentiation and engage overlapping downstream pathways (AKT and AMPK/ULK1-dependent autophagy), yet they differ in polarization potential: macrophages differentiated in IL-34 rather than CSF-1 secrete markedly more IL-10 under pro-inflammatory conditions and CCL17 under alternative-activation conditions^25^. Because IL-34 also signals through PTP-ζ (PTPRZ1) and syndecan-1 (CD138), and receptor usage was not resolved in that study, this divergence cannot be ascribed to CSF-1R engagement alone^26^. The polarizing output of a cytokine is set jointly by the receptor repertoire it engages and by the environment in which engagement occurs, rather than by ligand or receptor identity alone, since stromal IL-34 is required to maintain the perivascular macrophage pool and its loss perturbs microvascular function^27^.

The local metabolic environment is an equally powerful phenotypic determinant. Endothelial cells in ischemic muscle release lactate via MCT1, which drives macrophage polarization toward a pro-angiogenic state and rescues revascularization in pfkfb3-deficient models^28^—a striking example of endothelial-to-macrophage metabolic crosstalk directing vascular repair. In tumors, glutamine enrichment through GLS1-containing microvesicles promotes oxidative metabolism in macrophages, shifting them toward an angiogenesis-supporting phenotype in HER2+ gastric cancer^29^. These findings establish that tissue metabolic state is not merely a backdrop but an active instructor of macrophage angiogenic behavior.

Together, these findings reframe macrophage polarization in vascular biology as a continuously updated response to overlapping environmental inputs: cytokine gradients, metabolic signals, and paracrine cues from the vessel wall. The pro- or anti-angiogenic output of a macrophage population is therefore a property of its niche, a principle with direct implications for how macrophage-targeted therapies should be designed and timed in the context of microvascular regeneration.

## Mechanisms of macrophage-mediated microvascular angiogenesis

Macrophages regulate microvascular angiogenesis through four interlocking mechanisms: paracrine growth factor secretion, direct physical interactions with endothelial cells, proteolytic remodeling of the ECM, and crosstalk with mural cell progenitors. The following sections detail each mechanism, with emphasis on recently discovered pathways and molecularly defined macrophage subpopulations that execute them

### Paracrine growth factor secretion

**Fig. 2:**
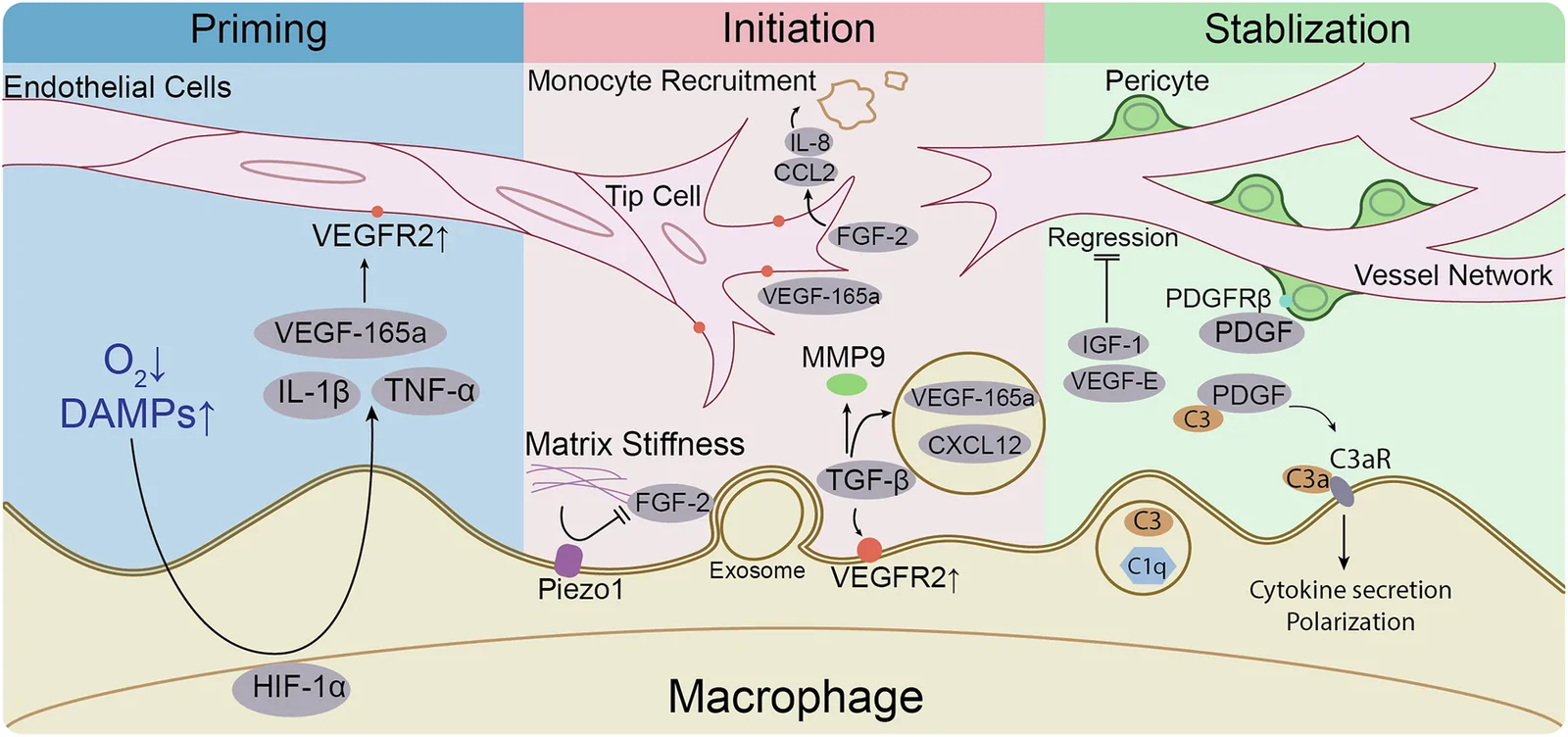
Mechanistic landscape of macrophage-endothelial crosstalk in vascular regeneration. The schematic is divided into 3 colour-coded zones corresponding to consecutive phases of the macrophage-derived paracrine program: priming(blue), initiation(pink) and stabilization(green). Throughout, the upper pink compartment represents the endothelium and the lower compartment represents the macrophage.

Macrophage-derived paracrine signaling is not a static cocktail of growth factors but a temporally ordered program carried out by macrophages of a variety of activation states. Upstream regulators like HIF-1α, TNF-α, and IL-1β sense the microenvironment and prime endothelial cells for response; initiation factors like VEGF, FGF2, and TGF-β drive tip-cell selection and sprouting; maturation factors like IGF-1 and PDGF recruit mural cells and stabilize nascent vessels (Fig. 2). The sections below treat each factor in that temporal order, emphasizing recently described mechanisms and the macrophage subsets that execute them.

#### Hypoxia-inducible factor-1 α (HIF-1 α)

HIF-1α functions as a master transcriptional integrator within macrophages, linking oxygen-sensing to pro-angiogenic gene expression. In zebrafish, macrophage-specific loss of HIF-1α impairs macrophage mobilization to injury sites and disrupts vascular repair^30^, demonstrating that hypoxia sensing in macrophages is required cell-autonomously for angiogenic responses. HIF-1α exerts these effects in part by inducing VEGF expression (cooperating with SMAD signaling as described above^31^) and by sustaining the pro-inflammatory macrophage activation state that drives early vessel sprouting. Beyond directly transactivating VEGF, macrophage HIF-1α also drives a hypoxia-responsive cyclooxygenase-2/prostaglandin E2/TNF-α axis that connects oxygen sensing to downstream inflammatory amplifiers, establishing HIF-1α as the operative integrator that sets both the priming (TNF-α/IL-1β) and the initiation (VEGF/FGF2) outputs of macrophage-driven angiogenesis^32^.

#### Tumor necrosis factor-alpha (TNF-α)

TNF-α is an early-acting macrophage-derived signal that primes the endothelium for angiogenic induction. In ex vivo aortic ring assays, macrophage-derived TNF-α precedes vessel sprouting; its genetic disruption impairs angiogenesis and reduces VEGF production, while exogenous TNF-α rescues these defects^33^. Macrophages are both source and sensor of TNF-α, creating an autocrine loop, while on the endothelial side TNF-α transcriptionally upregulates VEGFR-2 and neuropilin-1 via Sp1—not to initiate sprouting, but to raise the endothelium’s sensitivity to the VEGF signal that follows^34^. A second, paradoxical effect operates in parallel: while TNF-α upregulates VEGFR-2 transcription, it simultaneously blocks VEGFR-2 downstream signaling, likely via induction of the SHP-1 phosphatase^35^. This builds a reservoir of VEGFR-2 receptor protein on the endothelium that becomes signaling-competent only once the inflammatory phase subsides, providing a built-in temporal gate between macrophage-driven inflammation and VEGF-driven sprouting.

#### Interleukin 1β (IL-1β)

IL-1β is a central switch controlling both the magnitude and isoform specificity of macrophage VEGF output. By activating STAT3 and NF-κB at the VEGF-A promoter, IL-1β promotes transcription of the pro-angiogenic VEGF-A165a splice variant while suppressing VEGF-A165b^36^, representing a direct link of inflammatory modulation to angiogenic isoform balance. IL-1β and VEGF act synergistically in vivo: dual neutralization abolishes myeloid recruitment and capillary formation more completely than either alone^37^. A feedforward dimension is also established: macrophage-derived IL-1β sustains VEGF-C expression in the tumor microenvironment, with macrophage depletion reducing lymphangiogenesis and VEGF-C levels in tandem^38^. A 2025 study using a femoral artery ligation model with myeloid-specific VEGF depletion and subsequent inflammatory polarized macrophage transplantation demonstrated that the inflammatory IL-1β-expressing macrophages are sufficient to rescue blood flow^16^.

#### Vascular endothelial growth factor (VEGF)

VEGF is the most well-studied growth factor driving vessel growth, and macrophages have been shown to be a key cellular source of VEGF during angiogenesis. Macrophage VEGF output is not constitutive but tightly regulated by the inflammatory state of the niche. IL-1β drives transcription of the pro-angiogenic VEGF-A165a isoform via STAT3 and NF-κB, whereas loss of IL-1β signaling shifts expression toward the anti-angiogenic splice variant VEGF-A165b, impairing ischemic vascular recovery^15,16,36^. In a reciprocal loop, VEGFA-165b itself acts on macrophages to enforce a pro-inflammatory, anti-angiogenic phenotype via S100A8/S100A9–VEGFR1 signaling^24^—a feedback mechanism that can stall revascularization in peripheral artery disease. VEGFR1 signaling on macrophages also governs their recruitment to angiogenic sites, with bFGF acting downstream to amplify endothelial activation^39^. A particularly notable recent discovery is migrasome-mediated VEGF delivery. Embryonic monocytes patrolling future capillary-forming territory deposit membrane-enclosed migrasomes enriched in VEGFA and CXCL12 along their migration tracks; these structures are themselves sufficient to drive endothelial tube formation and recruit additional monocytes, identifying migrasome deposition as a spatially precise, stage-specific mode of angiogenic priming that is mechanistically distinct from diffusible factor secretion^40^. This positions monocyte migration routes as pre-patterned guidance cues for nascent vessel topology. Downstream of VEGF-mediated tip cell induction, macrophages act as cellular chaperones that physically bridge opposing endothelial sprouts to facilitate anastomosis^41^—a function addressed further in the direct cell contact section. Genetic deletion of VEGF-A specifically in myeloid cells reduces vascular density and disrupts vessel remodeling in tumors^42^, and paradoxically accelerates tumor growth, consistent with a shift from angiogenic initiation toward vessel stabilization as the dominant macrophage function at later stages.

#### Fibroblast growth factor-2 (FGF2)

FGF2(bFGF) exemplifies how physical properties of the microenvironment, not just chemical signals, control macrophage angiogenic output. A pivotal 2023 study demonstrated that macrophage Piezo1-mediated mechanosensation in stiff microenvironments suppresses FGF2 secretion via Ca²⁺/CaMKII-dependent inhibition of ETS1 transcriptional activity, with myeloid-specific Piezo1 knockout mice showing 2.3-fold higher FGF2 levels and improved perfusion recovery in hindlimb ischemia models^43^, establishing mechanical sensing as a direct upstream regulator of pro-angiogenic factor release. This has practical implications where the stiff fibrotic matrix that forms in chronic ischemia may suppress the macrophage FGF2 output needed for revascularization. In tumors, this mechanosensory brake is bypassed through transcriptional reprogramming. Hypoxia-induced lncRNA MALAT1 in tumor-associated macrophages (TAMs) upregulates FGF2 secretion, promoting endothelial tube formation and vasculogenic mimicry in thyroid cancer; silencing MALAT1 reverses these effects^44^. The relationship between FGF2 and macrophage recruitment is bidirectional: FGF2-stimulated endothelial cells secrete CCL2 and IL-8, generating chemotactic gradients that amplify monocyte infiltration^45^, while macrophage depletion substantially reduces FGF2-driven angiogenesis—a positive feedback loop that can sustain pathological neovascularization once initiated. Therapeutically, radiation-induced FGF2 polarizes TAMs toward immunosuppressive CD206⁺ phenotypes; anti-FGF2 treatment during radiotherapy repolarizes this balance and improves survival in murine tumor models^46^, identifying the Piezo1–FGF2–TAM axis as a candidate target for both ischemic repair, where relieving mechanical suppression may restore FGF2 output, and oncology, where blocking FGF2-driven TAM recruitment may normalize tumor vasculature.

#### Transforming growth factor-beta (TGF-β)

TGF-β coordinates angiogenesis through convergent actions on both macrophages and endothelial cells. Within macrophages, TGF-β was shown to induce VEGF-A expression through cooperative binding of Smad3/4 and hypoxia-inducible factor-1α/β (HIF-1α/β) at the VEGF promoter, with hypoxia potentiating this response^31^. TGF-β additionally upregulates fetal liver kinase-1 (Flk-1 or VEGFR2) on macrophages, a major VEGF receptor, and stimulates matrix metalloproteinase-9 (MMP-9) expression, coupling growth factor production and matrix remodeling within the same signaling program. By simultaneously driving VEGF secretion, receptor upregulation, and ECM remodeling, TGF-β functions as a master coordinator that primes the microenvironment for vessel sprouting. This primary angiogenic signal is further compounded by TGF-β-mediated macrophage polarization via SNAIL signaling (Section 2), establishing a feed-forward loop that amplifies the overall vascular response.

#### Insulin-like growth factor-1 (IGF-1)

Macrophage-derived IGF-1 regulates microvascular development across multiple tissue contexts. Wound macrophages express IGF-1 in vivo, with production amplified through CD44-hyaluronate interactions and dependent on TNF-α paracrine signaling^47,48^, linking the inflammatory phase of repair directly to angiogenic growth factor production. In the neonatal intestine, macrophage-derived IGF-1 is required for microvascular development: Cx3cr1-Cre-mediated IGF-1 deletion reduces intestinal microvascular density by 60% and triples the incidence of necrotizing enterocolitis, a finding that positions this axis as a physiologically essential, not merely modulatory, driver of organ vascularization^49^. Mechanistically, IGF-1 stabilizes nascent vessels by prolonging Erk activation relative to VEGF alone, counteracting lysophosphatidic acid–driven vessel regression in retinal and 3D endothelial models^50^, suggesting a role in the transition from active sprouting to structural consolidation of new capillaries. In tumor contexts, Tie2-expressing macrophages (TEMs) in ovarian cancer upregulate IGF-1 upon Angiopoietin-2 stimulation, driving endothelial tube formation via IGF-1R/Erk/Akt signaling; IGF-1 neutralization suppresses TEM-mediated angiogenesis and reduces metastasis in xenograft models^51^. IGF-1 therefore occupies a hinge position in the temporal program: it shares the sprouting-promoting activity of VEGF and FGF2 at early stages, then transitions to a stabilizing role by sustaining Erk signaling during the consolidation phase, distinguishing it from purely initiation-stage factors and qualifying it as the gateway to the maturation block discussed below.

#### Platelet-derived growth factor (PDGF)

In the context of microvascular angiogenesis, PDGF family members play distinct roles. PDGF-D promotes blood vessel maturation by enhancing macrophage recruitment and modulating interstitial fluid pressure. Co-expression with VEGF-E further stabilizes nascent vessels and reduces leakage^52^. Beyond vessel maturation, Macrophage-derived PDGF-D activates complement components C1q and C3 on macrophages, driving chemokine/cytokine release and polarizing macrophages into both pro- and anti-inflammatory phenotypes that collectively amplify choroidal neovascularization in age-related macular degeneration models^53^. Importantly, PDGF-BB secreted by macrophages also functions as the canonical chemoattractant for pericyte recruitment to nascent vessels, a function discussed further in the section on macrophage–mural cell crosstalk.

Across these growth factors, a recurring organizational principle emerges: macrophage-derived signals do not act independently but form an integrated, temporally staged program. TNF-α and IL-1β act early to sensitize the endothelium and determine VEGF isoform balance; VEGF itself, along with FGF2 and TGF-β, drives sprouting and tube formation; IGF-1and PDGF-D coordinate the later transition to vessel maturation and pericyte recruitment. Disruption of any individual factor shifts the balance of this program, with consequences that depend on the stage at which the disruption occurs. Understanding this temporal architecture is essential for designing interventions that promote functional, rather than merely structural, microvascular regeneration.

### Direct contact between macrophages and endothelial cells

**Fig. 3:**
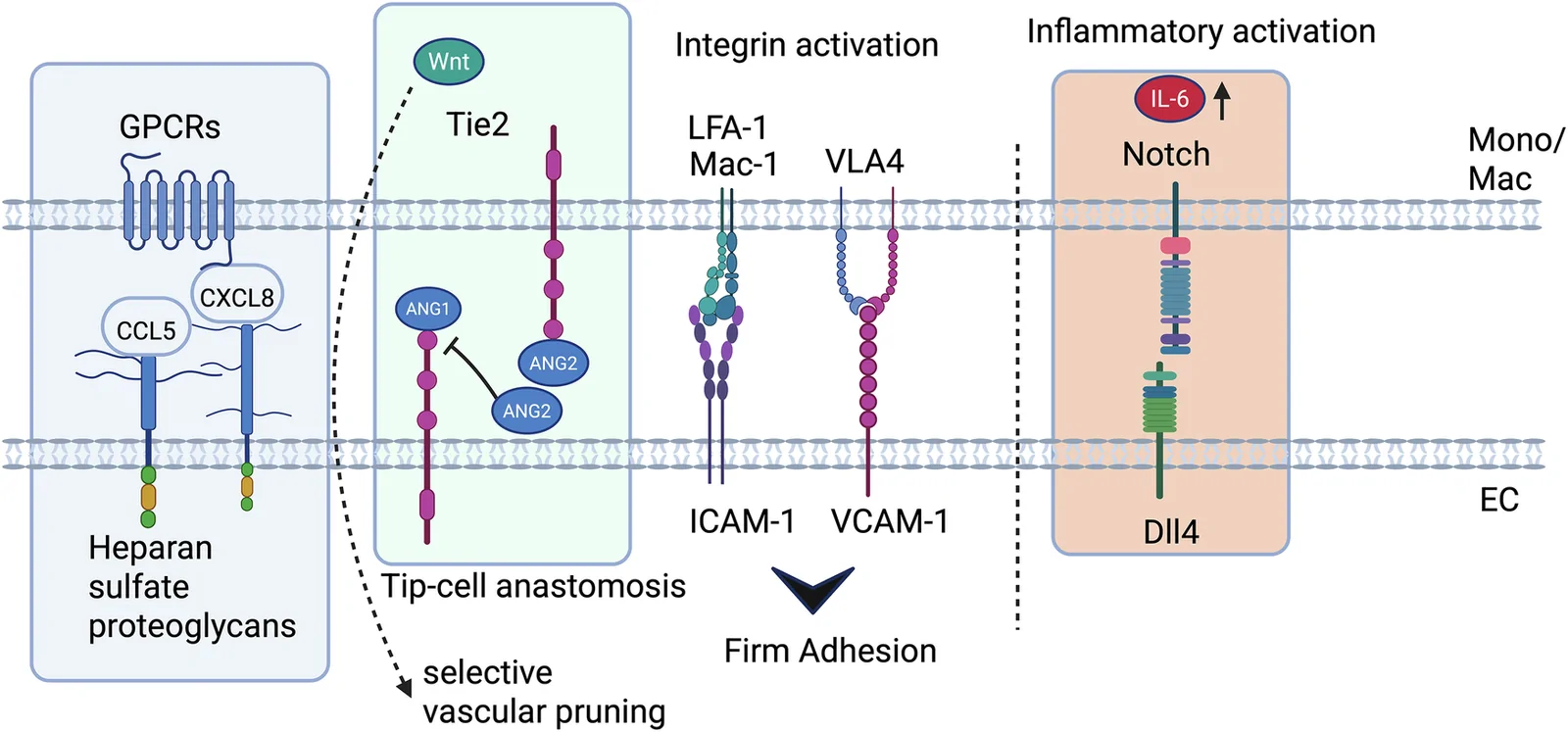
Macrophage EC direct contact during Angiogenesis. Created in BioRender. Huo, S. T. (2026) https://BioRender.com/qf4cutu.

Macrophages and endothelial cells communicate through direct, contact-dependent mechanisms that complement paracrine signaling during angiogenesis (Fig. 3). A review on all major adhesion molecules by Krieglstein highlighted that macrophage endothelial cell interactions are mediated through multiple complementary receptor–ligand pairs, including integrin–immunoglobulin superfamily interactions (β2-integrins/ICAM-1, ICAM-2; VLA-4/VCAM-1) and selectin-carbohydrate pairs (E-selectin, P-selectin/sialyl-Lewis X)^54^. These initial tethering and sustained firm adhesion are the first steps of monocyte/macrophage recruitment and retention at the endothelial surface before any angiogenic signaling.

TIE2-angiopoietin (ANG2) signaling represents one of the best-studied macrophage-endothelial interactions during angiogenesis. Mechanistically, endothelial-derived ANG2 engages TIE2 on proangiogenic macrophages, recruiting Tie2⁺ monocytes from bone marrow, enhancing their perivascular adhesion via upregulated integrins, and activating PI3K/AKT and MAPK pathways that drive proangiogenic polarization^55–57^. These macrophages in turn stabilize endothelial tip cells, secrete factors (VEGF, bFGF, PGE2), and physically bridge opposing sprouts to guide anastomosis, establishing a feedback loop essential for vessel maturation^18^. Genetic ablation of TIE2⁺ macrophages impairs revascularization in ischemic limbs, while macrophage exosomes delivering TIE2 amplify tumor angiogenesis^58^. Live imaging confirms the bridging role in zebrafish, macrophages migrate to fusion sites and spread laterally to align filopodia and sustain contact through lumenization; in murine hindbrain, they embrace fusing tip cells at junctions^30,41^.

This TIE2–ANG2 axis also coordinates vascular remodeling. Macrophages prune redundant vessels through phagocytosis of apoptotic endothelium, with depletion causing retinal hypervascularization^59,60^. Notably, ANG2 simultaneously destabilizes endothelial survival signaling while inducing macrophage Wnt production to trigger targeted regression, as seen in implant models where macrophages first wrap sprouting vessels, then engulf regressing segments^18,60,61^.

Beyond the TIE2/ANG2 axis, macrophages also engage endothelial cells through Notch/Dll4 juxtacrine signaling. Rather than simply promoting unchecked angiogenesis, the Notch/Dll4 acts as a restrictor for endothelial hypersprouting to allow proper vessel maturation. Dll4 signaling on endothelial tip cells regulates VEGF receptors on adjacent Notch-expressing cells, differentiating them into stalk cells, suppressing tip cell numbers. This pathway is counteracted by Jagged-tpye ligands, which promote sprouting by increasing tip cells^62^. Dll4-expressing endothelial cells in turn directly engage macrophages and drive them towards inflammatory polarization^63^.

Macrophage-endothelial direct interactions spatially and temporally coordinate angiogenesis through ligand-receptor crosstalk and physical bridging. Targeting these interactions has therapeutic potential for pathological angiogenesis in cancer or ischemic diseases.

### Macrophage and ECM remodeling in vascularization

Transcending their established roles as paracrine signaling hubs, macrophages function as physical “pathfinders” that mechanically and enzymatically deconstruct the ECM to facilitate endothelial invasion. This “bio-drill” mechanism was characterized by Moldovan et al., who described a form of neovascularization in the ischemic heart where monocytes and macrophages actively “drill” micro-conduits through the dense tissue matrix. Using MCP-1-overexpressing transgenic mice, researchers demonstrated that these inflammatory cells utilize metalloelastase (MMP-12) to create physical tunnels. These conduits serve as paths of least resistance that are subsequently colonized by endothelial cells to form functional, oxygen-delivering capillaries^64^.

MMP-9 secretion from macrophages extends this function beyond simple matrix clearance. By degrading gelatins and basement membrane collagens, MMP-9 not only removes physical barriers to migration but also unmasks cryptic regulatory sequences within type IV collagen that actively stimulate endothelial cell proliferation. Furthermore, macrophage-derived MMP-9 releases sequestered, matrix-bound VEGF, transforming the ECM from a passive scaffold into a reservoir of pro-angiogenic cues^65–67^.

Johnson et al. demonstrated that MMP-9-expressing macrophages localize specifically at perivascular capillary branch points during muscle ischemia. The physiological relevance of this localization was confirmed when bone marrow transplantation from wild-type donors rescued the impaired branching and decreased capillary density observed in MMP-9-null mice, establishing a paradigm where perivascular macrophage proteolysis directly dictates vascular architecture^66^.

The temporal window of angiogenesis is set by the balance between macrophage MMP output and endogenous TIMP inhibitors^68,69^. Future research must decode the molecular signals that polarize macrophages toward specific ECM-remodeling phenotypes. Mastering this “proteolytic rheostat” could enable targeted therapies to either invigorate revascularization in ischemic heart disease or suppress pathological angiogenesis in cancer and chronic inflammation.

### Macrophage crosstalk with mural cells in vessel maturation

A nascent endothelial sprout is not a stable vessel. Stabilization requires the recruitment of mural cells, pericytes, to the abluminal endothelial surface, where they suppress endothelial proliferation, deposit basement membrane, and confer mechanical integrity. Macrophages govern this final maturation step through three molecularly distinct routes: they recruit pericyte progenitors via PDGF-family chemoattractants, they drive progenitor differentiation through TGF-β activation, and in certain niches they serve as a direct cellular source of pericytes. The following sections detail each route and the macrophage subsets that execute it.

Two PDGF ligands recruit mural cells through distinct receptor routes. PDGF-BB, secreted by pro-angiogenic (CD206⁺) macrophages, engages PDGF receptor-β (PDGFRβ) on pericytes and is the canonical chemoattractant guiding their migration along the abluminal endothelial surface. Macrophages stimulated by IL4 and IL13 secrete the highest levels of PDGF-BB among macrophage subsets, potentiating its major role for pericyte secretion^70^. In parallel, a Lyve-1⁺ perivascular macrophage subset drives local proliferation of PDGFRα⁺ pericyte-like mesenchymal cells through a non-redundant PDGF-CC–dependent pathway, expanding the perivascular niche during tumor angiogenesis^20^; this represents a transcriptomically defined macrophage subpopulation with a dedicated mural-cell function. A third ligand, PDGF-D, enhances macrophage infiltration and downstream pericyte coating that reduces vascular leakiness^52^, linking the same macrophages discussed in the growth-factor section to mural-cell recruitment here.

Recruitment via the CXCL12–CXCR4 axis positions progenitors at the vessel wall, but their differentiation into functional mural cells requires macrophage-driven TGF-β activation. Minutti et al. defined this mechanism: macrophage-derived amphiregulin activates integrin-αV on perivascular mesenchymal cells, releasing bioactive TGF-β from its latent complex; the liberated TGF-β then drives pericyte differentiation toward a collagen-producing, vessel-stabilizing phenotype and restores vascular barrier function after injury^71^.

The macrophage-mural cell relationship is reciprocal. While macrophages recruit and differentiate pericytes, mural cells in turn sustain the perivascular macrophage pool: Van Hove et al. showed that IL-34 produced by mural cells and perivascular fibroblasts maintains brain perivascular macrophages, and that loss of stromal IL-34 alters the transcriptional state of vascular cells and perturbs microvascular function in pial and penetrating arterioles^27^. Beyond sustaining pericyte function through paracrine signals, macrophages can adopt mural cell identity directly, a finding that reframes their role in vessel stabilization from supportive to structural. Yamamoto et al. used fate-mapping with multiple transgenic mouse lines to show that at embryonic day E10.5, a subset of F4/80⁺ macrophages in the dorsal midbrain associates with nascent brain vessels and progressively acquires pericyte markers, demonstrating that a proportion of cerebrovascular pericytes originates from mature macrophages during the earliest phase of CNS vascular development^72^. This is not a transitional or intermediate state: the fate-mapped cells fully downregulate myeloid identity and take up residence in the perivascular wall as bona fide pericytes. In adult ischemic tissue, Amoedo-Leite et al. showed that this plasticity is recapitulated outside of development^73^. Single-cell RNA sequencing of fate-mapped macrophages from ischemic mouse muscle identified a subpopulation undergoing a transcriptional switch: downregulating canonical myeloid genes while upregulating mural cell markers including PDGFRβ, with RNA velocity analysis confirming active directional progression toward the mural cell state. The functional relevance was established by macrophage-specific PDGFRβ deletion, which abolished the perivascular macrophage phenotype, impaired vessel maturation, increased microvascular leakiness, and reduced limb perfusion after ischemia. Taken together, these findings reveal a direct macrophage-to-pericyte transdifferentiation that operates both in embryonic vascular development and in adult ischemic repair, and that is mechanistically separable from the paracrine PDGF-BB and amphiregulin/TGF-β routes described above.

## Conserved macrophage activity during development, wound healing and disease

Macrophages execute a conserved temporal program during microvascular angiogenesis that is recognizable across embryonic development, post-injury repair, and pathological neovascularization. An early inflammatory wave primes the endothelial cells and initiates sprouting. A phenotype switch follows, during which the macrophages transition to a resolution-phase state characterized by VEGF, TGF-β, PDGF, and IGF-1 output that supports vessel maturation, mural cell recruitment, and selective pruning^60,74–77^. The molecular triggers of each stage and the markers that define them are remarkably consistent across contexts. What differs is the boundary condition: developmental and reparative angiogenesis run the program to completion and terminate, whereas in disease the program either fails to switch or fails to resolve, producing the same machinery driving pathological rather than productive vascular outcomes.

The opening inflammatory wave is identifiable across all three contexts. In fetal organs, single-cell transcriptomics has identified a yolk-sac–derived perivascular macrophage population—distinct from microglia but sharing their primitive origin—that resides in the perivascular niche of multiple developing organs and expresses VEGFA, IL-1β, CXCL8, and TNF, the same canonical inflammatory–angiogenic signature deployed in post-injury repair^78^. In wound healing, this signature is induced acutely: damage-associated molecular patterns recruit and activate circulating monocytes that adopt a pro-inflammatory phenotype producing TNF-α, IL-1, IL-6, and IFN-γ within hours of injury^79,80^. In tumors, hypoxia produces an analogous IL-1β, HIF-α-responsive monocyte-derived TAM subset whose output dominates the peri-necrotic niche before being supplanted by other states^81^. The conservation of this initial wave—common upstream drivers (damage-associated molecular patterns, hypoxia, ANG2-mediated priming), common effectors (TNF-α, IL-1β, HIF-1α–driven transcription), and a common purpose (priming the endothelium for sprouting)—supports treating it as a single module rather than three context-specific phenomena.

The transition from this inflammatory wave to a resolution-phase state is the second conserved element. In embryonic vascularization, primitive macrophages progressively acquire pro-resolution functions as nascent vessels mature: yolk-sac–derived intestinal macrophages secrete IGF-1 that drives microvascular development and protects against necrotizing enterocolitis^49^, and primitive coronary macrophages selectively support perfused vasculature during coronary plexus remodeling^82^. In wound healing, the same switch defines the transition into the proliferative phase—macrophages shift to a state producing VEGF, TGF-β, and PDGF that supports endothelial maturation and pericyte recruitment, followed by a remodeling phase in which the same cells prune redundant capillaries^60,80^. In tumors, single-cell transcriptomics reveals TAM populations expressing CD206, ARG1, IL-10, and high VEGF—markers largely indistinguishable from those of post-injury resolution macrophages — that stabilize nascent vessels and produce the chronic angiogenic drive characteristic of solid tumor vascularization^83,84^. The same molecular switch operates in all three contexts; what varies is its kinetics and whether it terminates.

Disease emerges when this conserved program is interrupted, frozen, or chronically sustained, with two principal failure modes. The first is failure to switch out of the inflammatory phase: in diabetic and other non-healing wounds, macrophages remain locked in a pro-inflammatory, TNF-α– and IL-1β–producing state, sustaining tissue damage and preventing the proliferative and remodeling phases that would normally drive vessel maturation^85,86^. Metabolic and senescent dysregulation of macrophages in high-glucose environments has been mechanistically linked to this stalled switch and the resulting blunted angiogenic output. The second failure mode is failure to resolve: in solid tumors, macrophages execute the inflammation-to-resolution switch faithfully, but the resolution state is never terminated, producing a chronic VEGF/TGF-β/PDGF output that drives the leaky, disorganized, and incompletely matured microvasculature characteristic of tumor angiogenesis^81,87–91^. Both failure modes use the same molecular machinery as normal angiogenesis; the dysfunction is regulatory rather than mechanistic. Recasting impaired healing and tumor angiogenesis as endpoints of one conserved program—rather than as biologically distinct phenomena—clarifies why therapeutic strategies that target macrophage state (CSF1R inhibition, phenotype repolarization, HIF-1α blockade) are tractable across such different clinical contexts.

Across development, repair, and disease, then, the unifying observation is not that macrophages do different things in different contexts but that they execute the same temporally ordered program with different outcomes. The therapeutic implication is straightforward: rather than asking which macrophage subset to target in a given disease, the question becomes which stage of the conserved program is dysregulated and how to restore stage-appropriate signaling. Strategies that re-license stalled macrophages to advance through the program—biomaterial cues that promote the inflammation-to-resolution transition in chronic wounds, or pharmacologic interventions that withdraw tumor-associated macrophages from a chronic resolution state—operate on the same axis and validate the conserved-program framing as a unifying lens for vascular medicine.

## Clinical implications and therapeutic potential

Macrophages are increasingly recognized as a necessary component of vascular repair, and the clearest evidence comes from removing them. In a skin-graft model, clodronate depletion of adipose-derived microvascular fragments seeded onto collagen–glycosaminoglycan matrices reduced functional microvessel density and left surviving vessels wider, more slowly perfused, and less often covered by an α-SMA⁺ mural layer, implicating macrophages in vessel maturation as well as in network assembly^92^. These findings establish macrophages as required for vascularization, although they do not resolve which phase of the macrophage program mediates the effect. This requirement is task-specific. After obliteration of a single brain capillary endothelial cell, the flanking endothelial cells extend their membranes and restore continuity and flow independent of pericytes or glial cells^93^, mirroring the process extension by which neighboring pericytes re-cover denuded endothelium after single-pericyte ablation^94^. Macrophage input is required where a network must be built or expanded, rather than where continuity must be restored in an existing vessel. The same is true of efforts to build vasculature de novo: incorporating hPSC-derived primitive macrophages into heart-on-a-chip platforms restored long-term perfusability and primed stromal cells through IGFBP7 and HGF^95^, a translational advance that adds a third cell type to an engineered construct rather than correcting a transition in an existing population.

A second body of work has begun to treat the failed transition itself as the pathological target. Lineage tracing with Cx3CR1 reporter mice and bone marrow transplants showed that wound macrophages normally mature from a bone-marrow-derived, mixed-activation population into a long-term resident, alternatively activated one, and that it is the failure of this maturation step in diabetic mice that links persistent inflammation to delayed healing; the authors concluded that promoting maturation, rather than installing a terminal phenotype, may be the more effective therapeutic strategy in chronic inflammatory environments^96^. Biomaterials offer one route to this, as hydrogels can be tuned through stiffness, porosity, and surface chemistry to steer polarization, with recent work favoring temporally staged modulation over binary M1/M2 targeting^97^. The clearest vascular example is a protocatechuic aldehyde collagen hydrogel applied to diabetic wounds: without exogenous cells or added cytokines it reduced the M1 fraction of LPS-stimulated macrophages from 57.3% to 21.6% while raising the M2 fraction to 40.4% in vitro, lowered IL-1β and TNF-α while raising TGF-β and VEGF, and the macrophage-conditioned medium alone was sufficient to increase endothelial migration and tube formation; in diabetic rats this shortened the inflammatory phase, accelerated closure and produced more mature CD31⁺/α-SMA⁺ vessels^98^. The same logic, without a vascular readout, underlies pulmonary transplantation of IL-4-secreting macrophages, which sustained lung IL-4 for 48 h where injected cytokine decayed within 6 h and reduced injury and mortality by initiating the resolving phase early^99^. Pharmacological agents now achieve the same shift in resident cells. In diabetic wounds, topical low-dose aspirin shifted the wound macrophage population toward an anti-inflammatory, pro-resolving profile and accelerated wound closure, acting by promoting synthesis of the pro-resolving lipoxin LXA4 through the 5-LOX/LTA4/12-15-LOX axis while decreasing LTB4 and increasing efferocytosis; the benefit was abolished in mice lacking either 5-LOX or 12/15-LOX, tying it to the resolution pathway rather than to general anti-inflammation^100^. In glucocorticoid-impaired wounds, topical mineralocorticoid receptor antagonism likewise switched wound macrophages toward an anti-inflammatory phenotype, resolved the prolonged inflammation, upregulated pro-angiogenic factors and restored angiogenesis, accelerating healing—an effect reproduced by myeloid-specific deletion of the receptor, confirming the macrophage as the relevant target^101^.

Clinical translation is underway, though largely ahead of this mechanistic picture. Only a handful of registered trials are true macrophage cell therapies, the majority of them using ex vivo polarization and adoptive transfer, spanning cardiomyopathy, limb ischemia, stroke and arterial disease^102^. Intradural autologous M2 transplantation in 21 children with severe cerebral palsy proved safe over five years with sustained motor and cognitive gains^103^; no vascular or inflammatory endpoints were measured, but the trial establishes tolerability in humans. The stroke trial is more informative: intrathecal M2 delivery improved NIHSS in 75% of patients versus 18% of controls yet did not shift peripheral cytokine production overall, and responders had lower baseline IL-10, FGF-β, PDGF, and VEGF with higher pro-inflammatory stimulation indices than non-responders^104^, implying benefit accrued to patients who had not yet transitioned to resolution. Clinical advances are therefore real and accelerating; what is still missing is a validated way to identify which step is stalled in a given patient, though single-cell profiling of repair contexts is beginning to resolve the transitional states such a test would require.

## Conclusion

The picture that emerges from this body of work is not of a cell type that assists angiogenesis, but of one that architects it. Macrophages execute a temporally staged program—inflammatory priming, sprouting initiation, vessel maturation, and selective pruning—whose molecular logic is conserved from embryonic vascularization through wound repair and co-opted, without resolution, in tumor angiogenesis. Several recent findings sharpen this picture considerably. The discovery that monocytes deposit VEGFA- and CXCL12-enriched migrasomes along migration tracks reveals a mode of spatially encoded angiogenic instruction that diffusible secretion cannot provide. The Piezo1-mediated suppression of FGF2 in stiff matrices establishes the extracellular mechanical environment as a direct upstream regulator of macrophage pro-angiogenic output—with the practical implication that the fibrotic matrix of chronic ischemia may actively impair the macrophage response needed for repair. And the demonstration that a subset of perivascular macrophages downregulates myeloid identity and acquires PDGFRβ-dependent mural cell function in ischemic muscle positions macrophages not merely as recruiters of pericytes, but as a direct cellular source of vessel-stabilizing mural cells.

What remains unresolved is largely a question of resolution. The conserved-program framing presented here is built from studies that necessarily used broad macrophage markers, transcriptional snapshots, or bulk depletion models. The specific subpopulations that execute each stage—the cells that carry out the initial inflammatory priming versus those that license pericyte differentiation via amphiregulin—have been identified transcriptomically but not yet functionally isolated and perturbed in the same model system. It is not known whether the macrophage-to-mural cell switch described in ischemic muscle is a dedicated subpopulation with a predetermined fate, or an opportunistic response available to most perivascular macrophages under the right mechanical and metabolic conditions. Nor is it clear what terminates the program in normal repair but not in tumors—whether the difference lies in the macrophage itself, in the signals it receives from the vessel wall, or in the absence of a resolution-permitting metabolic transition.

The translational stakes of these questions are direct. Macrophage-based interventions for ischemic disease and vascular tissue engineering are advancing in the clinic, but without the ability to specify which macrophage state to deliver, when in the repair sequence, and to which vessel bed, their efficacy will remain empirical. Spatially resolved single-cell profiling of macrophage–vessel interactions across repair contexts, combined with genetic tools that allow stage-specific perturbation rather than wholesale depletion, will be necessary to move from conserved program to actionable target.

## Data Availability

Data sharing is not applicable to this article as no datasets were generated or analyzed during the current study.
